# Molecular and Physical Mechanisms of Fibrinolysis and Thrombolysis from Mathematical Modeling and Experiments

**DOI:** 10.1038/s41598-017-06383-w

**Published:** 2017-08-07

**Authors:** Brittany E. Bannish, Irina N. Chernysh, James P. Keener, Aaron L. Fogelson, John W. Weisel

**Affiliations:** 1 University of Central Oklahoma, Department of Mathematics and Statistics, Edmond, OK 73034 USA; 2 University of Pennsylvania School of Medicine, Department of Cell and Developmental Biology, Philadelphia, PA 19104 USA; 3 University of Utah, Departments of Mathematics and Bioengineering, Salt Lake City, UT 84112-0090 USA

## Abstract

Despite the common use of thrombolytic drugs, especially in stroke treatment, there are many conflicting studies on factors affecting fibrinolysis. Because of the complexity of the fibrinolytic system, mathematical models closely tied with experiments can be used to understand relationships within the system. When tPA is introduced at the clot or thrombus edge, lysis proceeds as a front. We developed a multiscale model of fibrinolysis that includes the main chemical reactions: the microscale model represents a single fiber cross-section; the macroscale model represents a three-dimensional fibrin clot. The model successfully simulates the spatial and temporal locations of all components and elucidates how lysis rates are determined by the interplay between the number of tPA molecules in the system and clot structure. We used the model to identify kinetic conditions necessary for fibrinolysis to proceed as a front. We found that plasmin regulates the local concentration of tPA through forced unbinding via degradation of fibrin and tPA release. The mechanism of action of tPA is affected by the number of molecules present with respect to fibrin fibers. The physical mechanism of plasmin action (crawling) and avoidance of inhibition is defined. Many of these new findings have significant implications for thrombolytic treatment.

## Introduction

Fibrinolysis, the enzymatic degradation of the fibrin mesh of blood clots, is mediated by plasmin, as is thrombolysis, the clinical process of thrombus dissolution by a lysis-enhancing drug. Plasmin degrades fibrin but *α*2-antiplasmin (*α*2-AP), a strong plasmin inhibitor, prevents appreciable plasma concentrations of free plasmin. Consequently, plasmin must be created locally on the fibrin fibers it is meant to degrade. Plasmin is formed when plasminogen and tissue-type plasminogen activator (tPA) co-localize on fibrin; after the formation of this “ternary complex,” tPA can convert plasminogen to plasmin^1^. Plasmin cuts fibers transversely^2–4^. This is likely due in part to the fiber configuration, which has binding sites located 6 nm apart transversely, and 22.5 nm apart longitudinally^5^. It has been proposed that the directed degradation across a fiber is due to plasmin’s ability to “crawl” to transverse binding sites, and inability to reach more distant binding sites^6^.

Fibrin clot structure is affected by the environment in which it forms^7^; importantly, high thrombin concentrations result in fine clots with thin, tightly packed fibers, while low thrombin concentrations result in coarse clots with thicker fibers and larger pores between fibers^8–10^. It is generally accepted that thick fibers lyse more slowly than thin fibers^2, 11^, but the literature offers conflicting observations. Some experiments show that coarse clots lyse more quickly than fine clots^12, 13^, while others show the opposite, or show no significant difference in lysis rates^14–16^. A single fibrin fiber is composed of many two-stranded protofibrils (long chains of half-staggered 45 nm-long fibrin monomers), aggregated laterally. Despite the seemingly tight packing of protofibrils into fibers, fibrin fibers are only about 20% protein and 80% water^17, 18^. Hence, many believe the fiber has pores through which small molecules can diffuse^19^, in addition to the larger pores between fibers in a clot.

To study the fibrinolytic process in a fibrin gel made of discrete fibers of varying thickness and pore size, we developed a 3D multiscale model^20^. Because of the low tPA concentration (plasma concentration 70 pM^19^, representative experimental or thrombolytic concentration 5 nM^2^) and high plasminogen concentration (plasma concentration 2 *μ*M^19^), we include both stochastic (random) and deterministic components in our model. We previously described^20^ some aspects of a prior version of such a model, but we have modified the model based on experiments described here to better capture the biology and examine clinically relevant scenarios (see Methods section and Supplement).

In this paper, we use this mathematical model of fibrinolysis to gain insight into several physical and mechanistic features of thrombolysis. To simulate clinical treatment situations, a bolus of tPA is introduced at the clot edge. We propose a general scheme for the effects of clot structure on clot degradation rates. We estimate rate constants that are necessary for a lysis front rather than uniform degradation. We provide a mechanistic explanation for why fibers are cut laterally rather than uniformly degraded. We suggest that, contrary to belief, plasminogen molecules are unable to diffuse through a single fibrin fiber. We determine the necessary conditions for plasmin to “crawl” as a processive enzyme. We conducted experiments to test certain model predictions. The effects of modifications to fibrinolytic drugs or dosing regimens can be investigated using this model.

## Methods

### Experimental procedures

Blood was obtained from healthy volunteers and all experiments were carried out in accordance with relevant guidelines and regulations and with approval from the Institutional Review Board of the University of Pennsylvania. Informed consent was obtained from all subjects. The blood was anticoagulated with trisodium citrate (1 vol of 0.13 M citrate for 9 vol of blood). Platelet-poor plasma was obtained by centrifugation of the blood samples at 10,000 g for 15 minutes; 0.10 mL of plasma was recalcified to a final concentration of 20 mM. Thrombin (American Diagnostica Inc, Stamford, CT) was added to initiate clotting, 0.1 U/ml final concentration for coarse clots and 1.0 U/ml for fine clots. For imaging, Alexa 488 labeled fibrinogen (Invitrogen, Carlsbad, CA) was added to the polymerization mixture at a final concentration of 0.03 mg/ml. After mixing, the sample was inserted into a glass chamber made from a microscope slide and a cover slip separated by a spacer made of two strips of double-stick tape. FITC-rtPA was prepared as previously described^2^ and these plasma clots were loaded with 5 *μ*L of single-chain rtPA (American Diagnostica Inc., Greenwich, CT USA) dissolved in platelet-poor plasma. Lysis experiments were imaged with a Zeiss 510LSM confocal microscope, using LSM software (Carl Zeiss, Thornwood, NY), equipped with a 63X1.4 NA Plan Apo objective lens, and lasers and filters for the two fluorophores. After 15 minutes of incubation in a moist atmosphere, the edge of the clot was imaged at regular intervals, and the lysis-front velocity of coarse and fine clots was measured at different rtPA concentrations (1, 2, 5, 10, and 20 nM).

### Multiscale model of fibrinolysis

**Figure 1:**
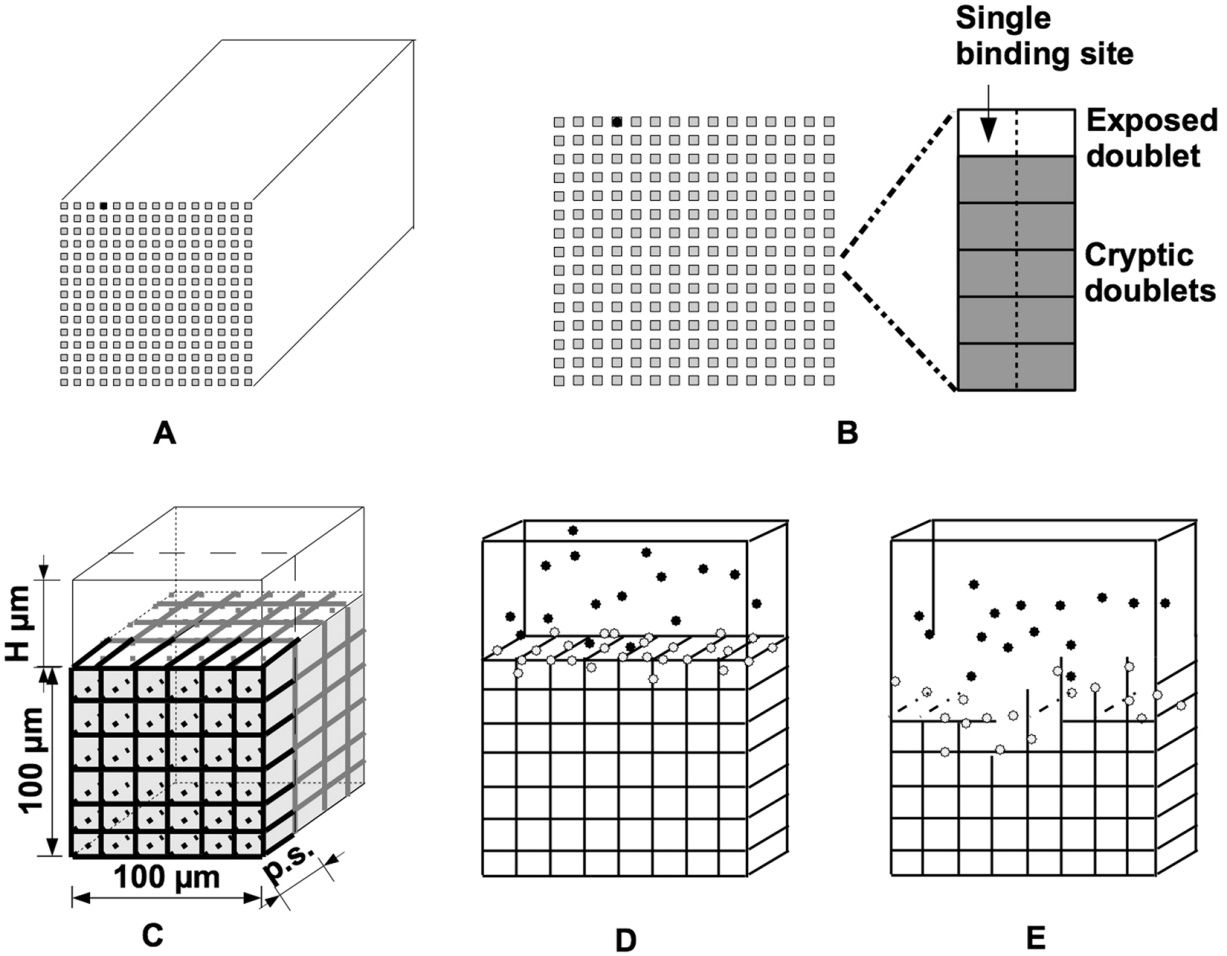
Schematic diagram of multiscale model. (**A**) The microscale model is a cross section (the square grid of gray squares) of the fibrin fiber. For simplicity, we approximate the circular cross section of the cylindrical fiber by a square of equal area. The microscale model is initialized with a single tPA molecule (black dot) randomly placed on the outer edge of the fiber. (**B**) On the left is the fiber cross section. Each gray square in the cross section represents 6 binding doublets (as seen on the right). A binding doublet is a pair of binding sites to which tPA, plasminogen, and plasmin can bind. Initially, there is one exposed binding doublet at each location, and 5 cryptic doublets that can be exposed by plasmin during degradation. A tPA molecule on a binding doublet may convert plasminogen on any doublet at the same binding location to plasmin. (**C**) The macroscale model represents a 3-D fibrin clot formed in a small chamber. The black and gray lines comprising the lattice represent the fibrin fibers. The size of the chamber is 100 × 100 × (100 + *H*) *μ*m^3^. For simplicity, we only consider one periodic slab of clot, one fiber thick (black). “p.s.” means “pore size”, the distance between fibers. (**D**) The macroscale model is initialized by randomly placing a fixed number of tPA molecules at the top of the chamber. The tPA molecules (black and white dots) can diffuse and bind to fibrin fibers (black). Unbound tPA is shown in black, bound tPA is shown in white. (**E**) When tPA binds to a fiber, it initiates the lytic process, so that some time after tPA has bound to a fiber, the fiber degrades.

The two-dimensional microscale model represents a single fiber cross-section (Fig. 1A,B), while the three-dimensional macroscale model represents a fibrin clot (Fig. 1C–E). Model variables include tPA, plasminogen, plasmin, and fibrin, which are tracked as they change in space and time. Motivated by the biology and clinical scenarios, we modified our previous multiscale model^20^. The microscale and macroscale domains were adjusted so that model fibrin concentrations were similar to physiological or clinical fibrin concentrations; the physical size of plasminogen was taken into account so that tPA-mediated activation of plasminogen and plasmin-mediated degradation of fibrin occurred on a more biologically reasonable spatial scale; and additional published parameter values were used (Table 1). Finally, the microscale data were input into the macroscale model in a more appropriate way. For details about how these changes were implemented mathematically in the new model, see Supplement. The model includes detailed chemical reactions, using all known rate constants, and realistic clot structures and protein concentrations. The model tracks individual tPA molecules as they interact with plasminogen and create plasmin, and it tracks individual plasmin molecules as they move across the fiber, expose new binding sites, and degrade fibrin. By solving the model equations, we learn how the amount of fibrin (represented by binding sites), tPA, plasminogen, and plasmin vary in time and space. We solve the equations with a hybrid method that uses the Gillespie algorithm^21, 22^. Results from the microscale model provide information about single fiber lysis times, the length of time tPA stays bound to a given fiber cross section, and the number of plasmin molecules created by a single tPA molecule. Due to the model’s stochastic nature, results from it vary even for a fixed set of parameter values. Hence, for each set of parameter values, we performed 10,000 microscale simulation experiments. From the resulting data, we compiled probability distributions for the tPA unbinding time and the single fiber degradation time, which we used in the macroscale model simulations, as described below. We considered only a fiber cross-section, rather than the whole fiber, since plasmin degrades fibrin by cutting across the fiber.

**Table 1:** Baseline parameter set used in the modified model.

Parameters	Value	Reference
*k*_deg_ (s^−1^)	5	39, 40
${k}_{{\rm{off}}}^{{\rm{PLG}}}$ (s^−1^)	3.8	41, 42
${k}_{{\rm{on}}}^{{\rm{PLG}}}$, intact fibrin (*μ*M^−1^ s^−1^)	0.1	41
${k}_{{\rm{on}}}^{{\rm{PLG}}}$, nicked fibrin (*μ*M^−1^ s^−1^)	1.72	42
${k}_{{\rm{c}}{\rm{r}}{\rm{a}}{\rm{w}}{\rm{l}}}^{{\rm{P}}{\rm{L}}{\rm{i}}}$ (s^−1^)	57.6	16
${k}_{{\rm{unbind}}}^{{\rm{PLi}}}$ (s^−1^)	0.05	43
${k}_{{\rm{off}}}^{{\rm{tPA}}}$, with plasminogen (s^−1^)	0.0002	44
${k}_{{\rm{off}}}^{{\rm{tPA}}}$, without plasminogen (s^−1^)	0.0036	45
${k}_{{\rm{on}}}^{{\rm{tPA}}}$ (*μ*M^−1^ s^−1^)	0.01	44, 45
${k}_{{\rm{cat}}}^{{\rm{ap}}}$ (s^−1^)	0.1	46, 47
${k}_{{\rm{cat}}}^{{\rm{n}}}$ (s^−1^)	5	—

${k}_{{\rm{on}}}^{{\rm{PLG}}}$ and ${k}_{{\rm{off}}}^{{\rm{PLG}}}$ are the binding rate constant for plasminogen to fibrin and the unbinding rate constant for plasminogen from fibrin, respectively, with dissociation constant ${K}_{{\rm{d}}}^{{\rm{P}}{\rm{L}}{\rm{G}}}={k}_{{\rm{o}}{\rm{f}}{\rm{f}}}^{{\rm{P}}{\rm{L}}{\rm{G}}}/{k}_{{\rm{o}}{\rm{n}}}^{{\rm{P}}{\rm{L}}{\rm{G}}}$. The tPA rate constants have similar meanings. ${K}_{{\rm{d}}}^{{\rm{P}}{\rm{L}}{\rm{G}}}$ is different depending on whether fibrin is intact or nicked, and ${K}_{{\rm{d}}}^{{\rm{t}}{\rm{P}}{\rm{A}}}$ is different depending on whether tPA is bound to a doublet with or without plasminogen. ${k}_{{\rm{cat}}}^{{\rm{ap}}}$ is the catalytic rate constant for activation of plasminogen to plasmin, ${k}_{{\rm{cat}}}^{{\rm{n}}}$ is the catalytic rate constant for plasmin-mediated exposure of cryptic binding doublets, and *k*_deg_ is the plasmin-mediated rate constant for fibrin degradation. ${k}_{{\rm{c}}{\rm{r}}{\rm{a}}{\rm{w}}{\rm{l}}}^{{\rm{P}}{\rm{L}}{\rm{i}}}$ and ${k}_{{\rm{unbind}}}^{{\rm{PLi}}}$ are the crawling rate constant and unbinding rate constant of plasmin, respectively. We know the dissociation constants for tPA and plasminogen, but not the individual rate constants, so references in the table are for *K*_d_. We assume that ${k}_{{\rm{cat}}}^{{\rm{n}}}={k}_{\deg }$, since we have not found any references for the rate of plasmin-mediated exposure of binding sites.

Data from the microscale model were used in the macroscale model of a fibrin clot, from which we obtained lysis front velocities and tPA distributions. The macroscale model domain is a 3-D square lattice representing a clot; each lattice edge represents a fibrin fiber. Different clot structures are explored by changing the width of lattice edges (fiber diameter) and the distances between edges (clot pore size). In each macroscale simulation, a tPA bolus is added to a fibrin-free region abutting the clot, and each tPA molecule is allowed to diffuse and bind to/unbind from fibers. When a tPA molecule binds to a fibrin fiber on the macroscale, it initiates the lytic cascade on the microscale. Probability distributions obtained from microscale simulations are used to determine when the tPA molecule unbinds on the macroscale and when the fiber degrades. In this way, the biochemistry only appears implicitly in the macroscale model, via results from the microscale model. The model most closely represents *in vitro* experiments, but is also used to model thrombolysis of a fully occluded blood vessel. As such, we do not consider blood flow in the model.

## Results

### Lysis rate is determined by the interplay of tPA and clot structure

Results from the many studies on factors affecting fibrinolysis have been conflicting. Mathematical models closely tied with experiments can be used to understand relationships within the complex fibrinolytic system. We varied fiber diameter and clot pore size in our model to simulate fine clots containing thin fibers and small pores, and coarse clots containing thick fibers and large pores, both composed of the same total amount of protein. To simulate typical clots, fine clots contained 97.5-nm-diameter fibers spaced 1.37 *μ*m apart, and coarse clots contained 195-nm-diameter fibers spaced 2.74 *μ*m apart, although these parameters are easily varied. The parameter values used here are based on measurements from scanning electron microscopy and quantitative analysis of confocal microscope images. We initialized the mathematical models with boluses of varying amounts of tPA by changing the volume and/or concentration of tPA solution added.

**Figure 2:**
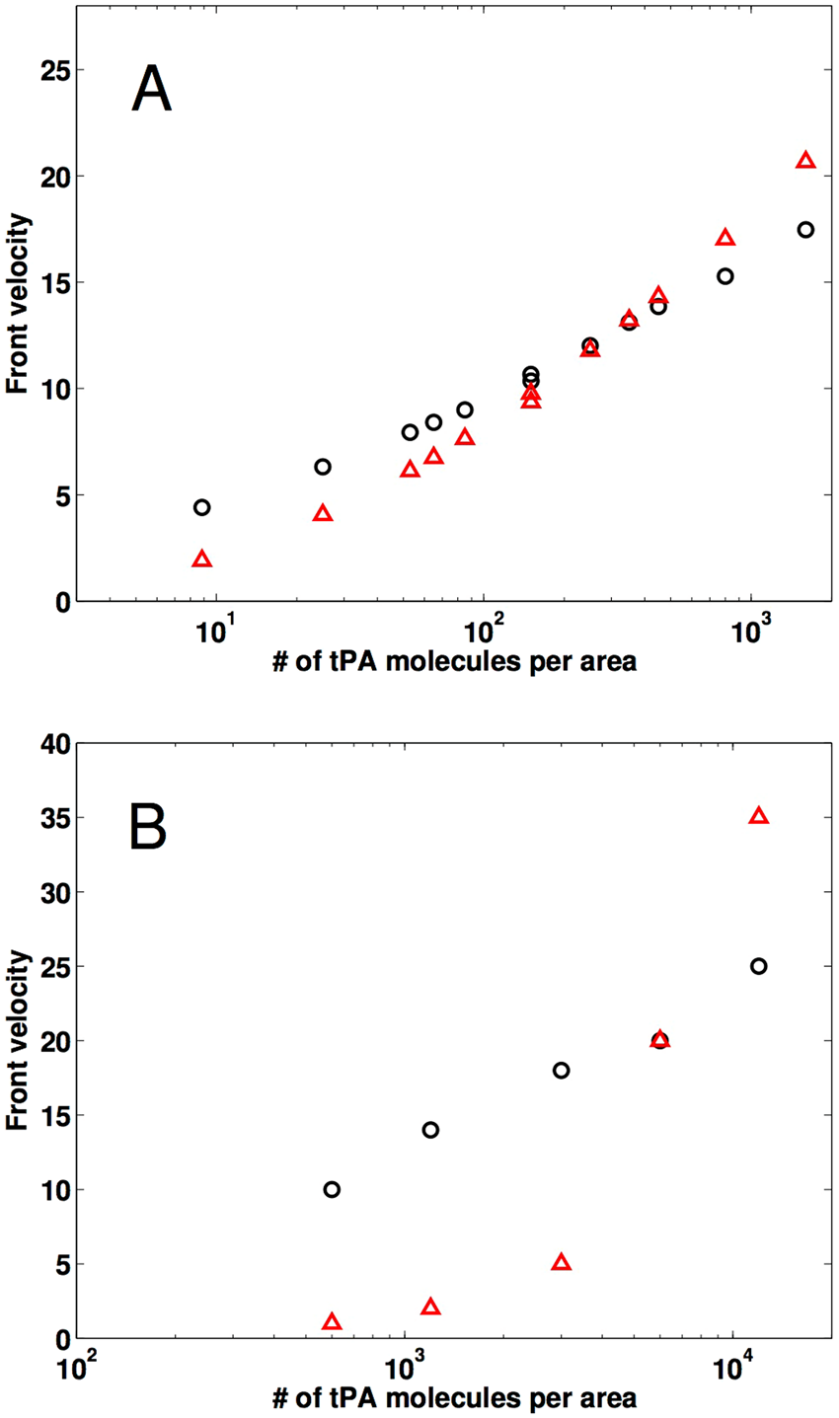
Lysis front velocity as a function of clot structure and tPA concentration. Lysis front velocity, in *μ*m/min, of a fine clot (red triangles) and a coarse clot (black circles) as a function of the ratio of the number of tPA molecules to the surface area of clot exposed to the fibrin-free region. (**A**) Mathematical model simulations were run with 11 different tPA-to-surface-area ratios varying from 8 to 1600 molecules/*μ*m^2^. Each symbol indicates the mean of ten independent simulations. For a tPA-to-surface-area ratio of 150, a pair of distinct experiments were run for each clot type, one with an 85 nM tPA solution and the other with a 5 nM tPA solution, and the results for each pair were almost the same (as seen by the double symbols). (**B**) Laboratory experiments were run with 5 different tPA-to-surface-area ratios varying from 600 to 12000 molecules/*μ*m^2^. Each symbol indicates the mean of three independent trials. Clots were formed from the pooled plasma of 6 donors.

We used our mathematical model to elucidate the factors that influence lysis speeds in clots of varying structure and thereby identified a mechanism that can explain the conflicting results in the literature: the number of tPA molecules relative to the surface area of the clot exposed to those molecules (in conjunction with the number of fibers in the clot), determines whether fine clots lyse faster or slower than coarse clots (Fig. 2A). Thus, at low tPA concentrations coarse clots are lysed more rapidly but at high tPA concentrations fine clots are lysed faster. This same qualitative pattern is seen in *in vitro* plasma clot experiments (Fig. 2B).

As expected, lysis rates increase with an increasing amount of tPA. By fitting a line to the data (fine clot velocity)/(coarse clot velocity) and solving for the number of tPA molecules per area at which this ratio equals 1, we used the mathematical model to estimate 323 molecules/*μ*m^2^ to be the tPA-to-surface-area ratio below which coarse clots lyse faster than fine. Note that it is the *number* (concentration times volume), not concentration alone, of tPA that determines relative lysis speed.

To verify that it is the number of tPA molecules that determines clot lysis velocity, we ran two simulations in which the number of tPA molecules/*μ*m^2^ was fixed at 150. In the first simulation, we added 85-nM tPA solution to fill a height of 2.94 *μ*m above the surface of the clot. In the second, we added 5-nM tPA solution to fill a height of 49.9 *μ*m above the surface of the clot. These experimental conditions are similar to the clinical situation of a bolus of tPA adjacent to a thrombus. The two situations gave essentially identical results (Fig. 2A). Since there was little variation in lysis rate when substantially different volumes and concentrations of tPA were used, we conclude that it is the number of tPA molecules that determines the relative lysis speed.

### Kinetic conditions necessary for fibrinolysis or thrombolysis to proceed as a lysis front

The mathematical modeling here has all been done using the best kinetic parameters available in the literature. However, there are only estimates of binding and unbinding rate constants for fibrinolytic proteins^14^, because measuring these rate constants is difficult^23^. We used our model to investigate how lysis depends on binding and unbinding rate constants (for fixed dissociation constants) and to estimate a range of plausible parameter values (Table 2

**Table 2:** Model results for varying binding and unbinding rate constants.

Parameters	Fiber diameter, nm	Forced unbind	Runs with plasmin	Front velocity, *μ*m/min
Baseline	97.5	4760	5856	1.97 ± 0.10
	195	4895	5892	4.40 ± 0.24
Small tPA rate constants${k}_{{\rm{on}}}^{{\rm{tPA}}}$ = 1 × 10^−4^ *μ*M^−1^ s^−1^, ${k}_{{\rm{off}}}^{{\rm{tPA}}}$ (without PLG) = 3.6 × 10^−5^ s^−1^, ${k}_{{\rm{off}}}^{{\rm{tPA}}}$ (with PLG) = 2 × 10^−6^ s^−1^	97.5	8316	9935	—
	195	9054	9939	—
Large tPA rate constants${k}_{{\rm{on}}}^{{\rm{tPA}}}$ = 1.0 *μ*M^−1^ s^−1^, ${k}_{{\rm{off}}}^{{\rm{tPA}}}$ (without PLG) = 0.36 s^−1^, ${k}_{{\rm{off}}}^{{\rm{tPA}}}$ (with PLG) = 0.02 s^−1^	97.5	37	148	1.67 ± 0.09
	195	43	145	2.43 ± 0.10
Intermediate PLG rate constants${k}_{{\rm{on}}}^{{\rm{PLG}}}$ (intact) = 1 × 10^−3^ *μ*M^−1^ s^−1^, ${k}_{{\rm{on}}}^{{\rm{PLG}}}$ (nicked) = 1.72 × 10^−2^ *μ*M^−1^ s^−1^, ${k}_{{\rm{off}}}^{{\rm{PLG}}}$ = 0.038 s^−1^	97.5	2729	3406	0.75 ± 0.28
	195	2722	3369	3.04 ± 0.23
Small PLG rate constants${k}_{{\rm{on}}}^{{\rm{PLG}}}$ (intact) = 1 × 10^−4^ *μ*M^−1^ s^−1^, ${k}_{{\rm{on}}}^{{\rm{PLG}}}$ (nicked) = 1.72 × 10^−3^ *μ*M^−1^ s^−1^, ${k}_{{\rm{off}}}^{{\rm{PLG}}}$ = 0.0038 s^−1^	97.5	1126	1413	0.52 ± 1.38*
	195	1061	1367	1.78 ± 0.36

“Baseline” corresponds to parameter values in Table 1, and other parameter values differ from baseline values only as indicated. The column labeled “Forced unbind” gives the number of runs out of 10,000 microscale simulation runs for which tPA was forced to unbind by plasmin. The column labeled “Runs with plasmin” gives the number of microscale runs, out of 10,000, in which plasmin was produced and single fiber lysis occurred. For the macroscale model results (“Front velocity”), the experiments involved a 5 nM tPA concentration added to a fibrin-free region with height 2.935 *μ*m. Entries are mean ± standard deviation of 10 independent simulations. Front velocity calculations do not make sense for simulations in which lysis was not front-like, hence the “−” in some entries. An asterisk indicates a case in which lysis is front-like (tPA strongly binds to the front), but so slow that it is computationally challenging to measure front velocity.

). We discarded values that gave results contrary to experimental evidence (e.g., very slow or very fast clot lysis rates, or lysis that is not front-like), and kept values that resulted in reasonable lysis. For thoroughness and to test the mathematical model, we examined a wide range of values, including values that may be unlikely physiologically. As we expect (and as discussed below), for the non-physiological rate constants the model fails to predict front-like lysis with a reasonable front velocity.

It has been observed both *in vitro* and *in vivo* that when tPA is introduced at the edge of a clot or thrombus, lysis proceeds spatially as a front advancing with time. However, it is not intuitively obvious why this is so. It is possible that tPA could diffuse into the clot and then bind to fibers, such that digestion would then not progress as a front but would occur simultaneously throughout the clot. With our model we investigated the conditions necessary for front-like behavior. The practical implications of these conclusions are that variants of tPA and/or plasminogen could be designed that would alter this behavior; with modified proteins, the lysis front velocity could be increased or lysis could be made to occur uniformly, simultaneously, and more rapidly.

With tPA binding and unbinding rate constants that are 2 orders of magnitude smaller (“Small tPA rate constants”) than the baseline values from Table 1, lysis was not front-like. Due to its small binding rate to fibrin, tPA was able to diffuse through the clot easily, starting degradation on many fibers throughout the clot, and was not constrained to a “lysis front”. When tPA binding and unbinding rate constants 2 orders of magnitude larger than baseline values were used (“Large tPA rate constants”), tPA quickly unbound from the fiber cross-section and consequently single-fiber lysis occurred in only about 1.5% of the 10,000 microscale model simulations. Although single-fiber degradation was impeded, macroscale lysis still proceeded in a front-like manner and with reasonable lysis front velocities.

With plasminogen binding and unbinding rate constants 2 orders of magnitude smaller than baseline values (“Intermediate PLG rate constants”), lysis front velocities were reasonable. However, with plasminogen binding and unbinding rate constants 3 orders of magnitude smaller (“Small PLG rate constants”), macroscale lysis was slowed considerably, to the point that it was computationally difficult to measure the lysis front velocity. Other scenarios have also been considered, e.g. binding/unbinding rate constants that increase the lysis front velocity or conditions under which lysis does not proceed in a front-like manner, but is more rapid.

### Plasmin regulates the effective local tPA concentration

Planning thrombolysis requires that sufficient plasminogen activation be present for effective lysis but not so much that it can cause bleeding elsewhere. Thus, it is important to know not only the total tPA introduced but also the tPA released from the digesting clot. Using the microscale model, we estimated the length of time tPA stays bound to a fiber. There are two ways in which tPA is released from fibrin: simple unbinding (via the kinetic unbinding rate), and forced unbinding due to plasmin-mediated degradation of fibrin. Plasmin does not necessarily degrade the actual tPA binding site, but rather cleaves the fibrin to which tPA is bound. tPA may remain bound to this fibrin degradation product, but it is no longer part of the fibrin fiber, and therefore we consider it to have been forcibly unbound from the fiber.

**Figure 3:**
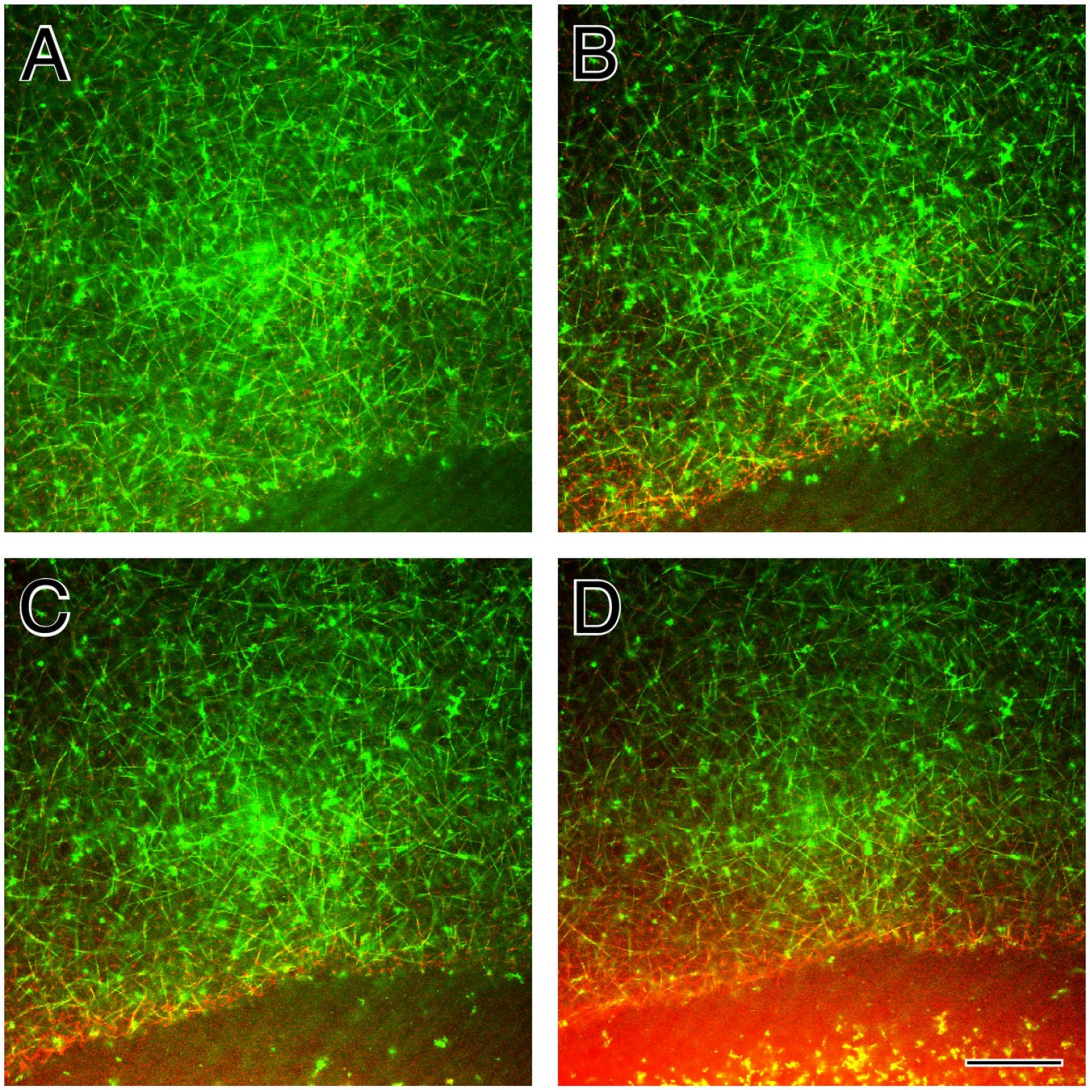
Confocal micrographs of a fibrin clot during fibrinolysis initiated by tPA. Fluorescently labeled fibrin is green, tPA is red, and the overlap of fibrin and tPA thus makes yellow. Micrographs are (**A**) initial image, (**B**) 147 sec later, (**C**) 196 sec later, (**D**) 245 sec later. Magnification bar = 5 *μ*m.

Experiments show tPA released from the clot by digestion in the area adjacent to the clot (Fig. 3). Fluorescently-labeled fibrin (green) and tPA (red) are visualized by confocal microscopy as lysis proceeds; the digested clot pieces are yellow due to the overlap of tPA and fibrin (Fig. 3D). This supports the prediction that fibrin-bound tPA can be removed from the clot via fibrin degradation.

For baseline parameter values (Table 1), the model predicts that tPA is forced to unbind by plasmin-mediated degradation of fibrin about 48% of the time (Table 2). Except for the most extreme range of parameter values tested, tPA is forced to unbind over 10% of the time (Table 2). So plasmin directly affects the ability of tPA to move through a clot; the more plasmin in the system, the sooner tPA will be forced to unbind from a given fiber and be able to diffuse to a neighboring fiber. In this way, plasmin can regulate the effective diffusion of tPA through a clot.

### The mechanism of action of plasmin is affected by the number of tPA molecules present per fibrin fiber

In experiments that inject a 5-nM bolus of tPA, there are only 3 tPA molecules per cubic micron in the clot; a physiological concentration of tPA (70 pM) amounts to 0.04 molecules/*μ*m^3^ (Supplement). For context, the length of a tPA molecule in solution is about 10 nm^24^. Assuming the shape of tPA to be spherical (to simplify calculations), the volume of a single tPA molecule is 5.24 × 10^−7^ *μ*m^3^. So over 1 million tPA molecules could fit in a 1 *μ*m^3^ volume. In a plasma clot, which has a characteristic pore size of 1 *μ*m, the volume enclosed by nearest-neighbor fibers is approximately 1 *μ*m^3^. Since a 5 nM tPA concentration amounts to just 3 tPA molecules per cubic micron, it is likely that a fibrin fiber will encounter only a single tPA molecule. The presence of tPA in discrete spots in confocal images of digesting clots (Fig. 3A–C, red fluorescence) shows the sparsity of tPA. Thus, a single fibrin fiber may only encounter a small number of tPA molecules, even in the conditions of thrombolytic therapy.

### The average space between protofibrils limits the physical accessibility of the fiber interior to macromolecules such as plasmin(ogen)

**Figure 4:**
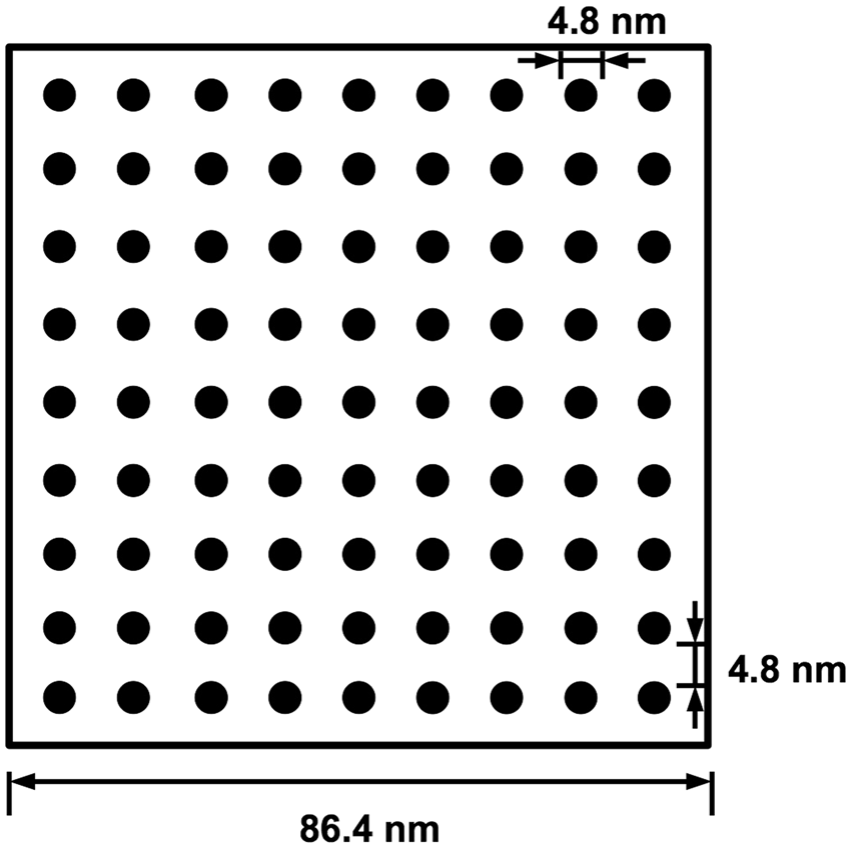
Diagram of protofibril spacing in a fiber cross section. The circular fiber cross section of a 97.5-nm diameter fiber is approximated by a square of equal area. Black circles are protofibril cross sections, each with diameter 4.8 nm. The distance from the edge of one protofibril to the edge of a neighboring protofibril is also 4.8 nm.

A calculation of the average space between protofibrils (Supplement) shows an edge-to-edge distance between protofibrils of approximately 4.8 nm (Fig. 4). Hence, a plasminogen molecule with diameter approximately 9–11 nm^25, 26^, is unable to diffuse through the “holes” between protofibrils.

### The physical mechanism of plasmin action (crawling) and avoidance of inhibition

**Figure 5:**
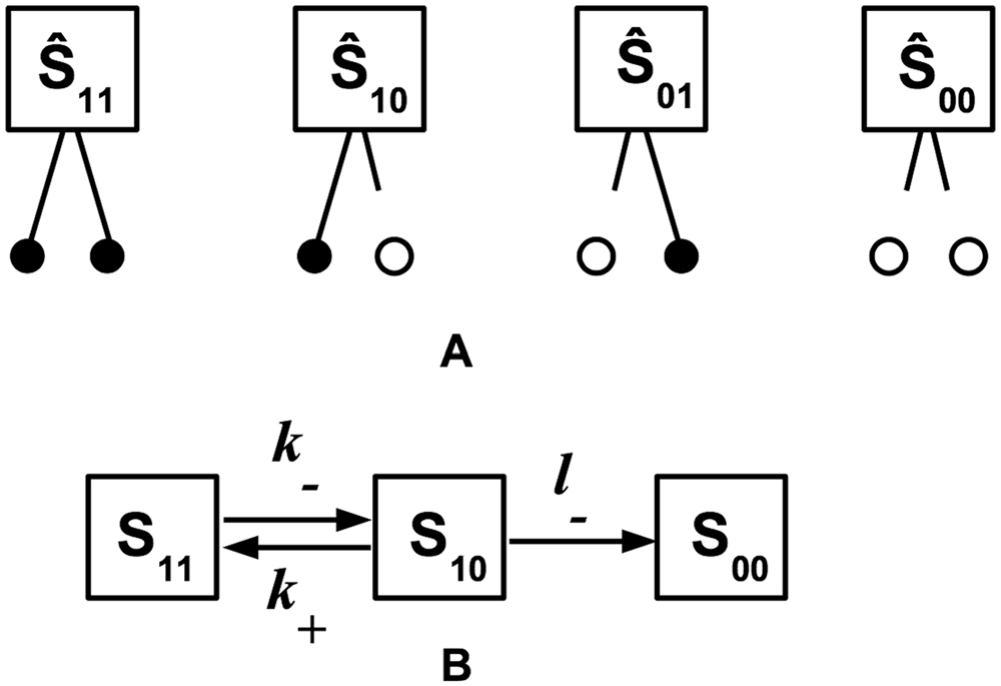
Basic model for plasmin crawling. (**A**) The 4 possible states a plasmin molecule with 2 limbs can be in. A filled circle represents fibrin with a plasmin limb bound and an open circle represents fibrin with a plasmin limb unbound. (**B**) Reaction diagram for a plasmin molecule transitioning between states. We redefine variables such that *S*_00_ = $\hat{{\rm{S}}}$_00_ (which represents all states with two unbound limbs), *S*_11_ = $\hat{{\rm{S}}}$_11_ (which represents all states with 2 bound limbs), and *S*_10_ = $\hat{{\rm{S}}}$_10_ + $\hat{{\rm{S}}}$_01_ (which represents all states with 1 bound and 1 unbound limb). The possibility of an unbound plasmin (*S*_00_) binding is neglected since we are interested in what happens prior to the plasmin molecule unbinding. Since it is unlikely for two limbs to unbind at the *exact* same time, we assume that *S*_11_ cannot transition directly to *S*_00_, but must first transition to *S*_10_.

Plasma-phase plasmin molecules are quickly inhibited by *α*2-AP^27^, while fibrin-bound plasmin is protected from *α*2-AP^28, 29^. A plasmin molecule contains multiple lysine-binding kringles which allow it to bind to fibrin and perform its fibrinolytic function without being inhibited. Specifically, kringle 1 (K1) contains the primary lysine binding site for plasmin(ogen) to fibrin, and kringle 4 (K4) reinforces the binding^25, 30^. Therefore, a plasmin molecule is thought to have a proteolytic “head” domain for proteolysis and two “limbs” (the K1 and K4 kringles) for binding to fibrin^6^. Analysis of a simple mathematical model of a plasmin molecule with two “limbs” it can use to crawl (Fig. 5) shows that the expected number of “steps” before the molecule unbinds from fibrin (and becomes susceptible to *α*2-AP) is given by the ratio ${k}_{{\rm{c}}{\rm{r}}{\rm{a}}{\rm{w}}{\rm{l}}}^{{\rm{P}}{\rm{L}}{\rm{i}}}/{k}_{{\rm{u}}{\rm{n}}{\rm{b}}{\rm{i}}{\rm{n}}{\rm{d}}}^{{\rm{P}}{\rm{L}}{\rm{i}}}$ (Supplement). Here ${k}_{{\rm{c}}{\rm{r}}{\rm{a}}{\rm{w}}{\rm{l}}}^{{\rm{P}}{\rm{L}}{\rm{i}}}$ is the rate at which plasmin crawls and ${k}_{{\rm{u}}{\rm{n}}{\rm{b}}{\rm{i}}{\rm{n}}{\rm{d}}}^{{\rm{P}}{\rm{L}}{\rm{i}}}$ is the kinetic unbinding rate of plasmin from fibrin (both in units s^−1^). For a plasmin molecule to crawl, it must take many “steps” before it unbinds and is inhibited by *α*2-AP; this can only be achieved if the crawling rate is much larger than the unbinding rate.

## Discussion

**Figure 6:**
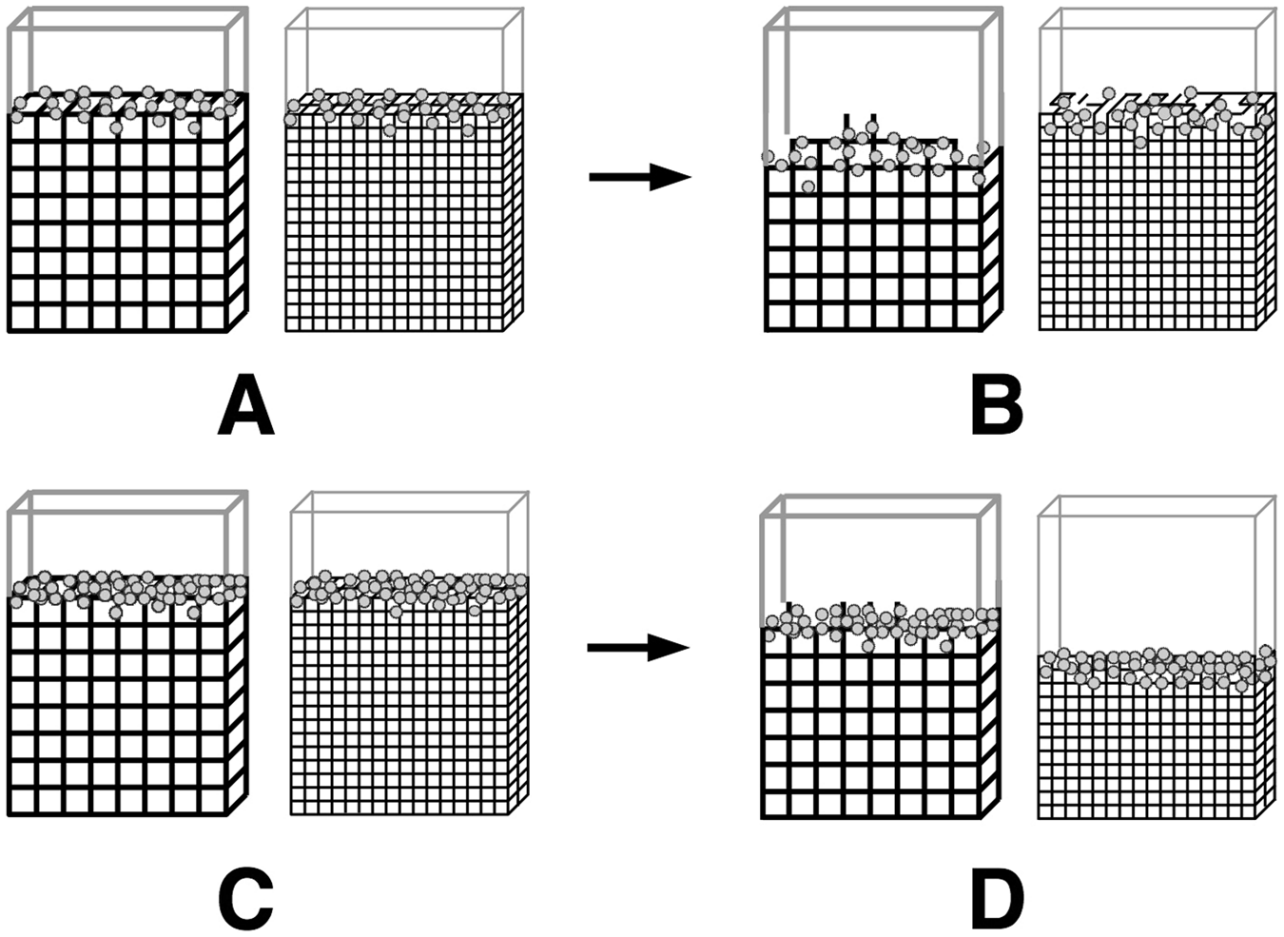
Schematic of the interplay between clot structure and tPA. Each subfigure depicts a coarse clot with thick fibers (left) and a fine clot with thin fibers (right) contained in the same volume. Round symbols represent bound tPA molecules. (**A**,**B**) When there is not much tPA present, the coarse clot degrades faster because it has fewer fibers; the small number of tPA molecules is able to start degradation on all fibers at the coarse clot front, but is unable to do so at the fine clot front. (**C**,**D**) When there is a lot of tPA present, degradation begins on all fibers at the front of both clot types. In this case, the fine clot degrades faster since the individual thin fibers in the fine clot are lysed faster than the individual thick fibers in the coarse clot.

Our mathematical model accounts for previously conflicting experimental results and predicts the effects of different dosing regimens and molecular modifications of tPA or plasminogen on thrombolysis. The model predicts how the lysis rate depends on clot or thrombus structure and the amount of tPA present; it is the *number of molecules*, not concentration alone, of tPA that determines relative lysis speed. Because tPA binds strongly to fibrin, tPA molecules quickly accumulate at the clot front. So 1.0 mL of 5-nM tPA solution results in similar lysis as 0.1 mL of 50-nM tPA. The fast diffusion and strong binding of tPA quickly localize the molecules to the clot front. This accounts for the complex relationship between clot structure and lysis rates. With a large number of tPA molecules, there is enough tPA to start lysis on all fibers at the lysis front in both fine and coarse clots. Since the thin fibers in the fine clot lyse faster than the thick fibers in the coarse clot, the fine clot degrades more quickly (Fig. 6C,D). However, with a small number of tPA molecules, there is enough tPA to start lysis on all fibers at the lysis front of the coarse clot, but there is only enough tPA to start lysis on some fibers at the lysis front of the fine clot. Although it takes longer for each thick fiber to degrade, the coarse clot has an advantage since it contains fewer total fibers (Fig. 6A,B). These results may have important implications for thrombolysis, since thrombi in different vessels (coronary arteries^31, 32^, cerebral arteries^33^, veins^34^) have different structures and composition. These results also have applicability to dosing regimens of thrombolytic agents. While a more complicated model that takes into account cellular components of thrombi and patient-specific parameter values would be required to directly set dosing regimens for clinical use, general implications for thrombolysis can be obtained from the current model. For example, administering a higher dose of tPA could be beneficial for a patient with a tightly-packed clot, but would have little positive effect for a patient with a coarser clot. In fact, increasing the tPA dose may be detrimental to patients with coarser clots, as the additional tPA could contribute to systemic bleeding.

Mathematical modeling makes it possible to vary all parameters, so different ideas can be tested and compared to determine properties that could be most beneficial for new thrombolytic agents. Besides tPA release at the kinetic unbinding rate, tPA is also forced to unbind by plasmin-mediated degradation of the fibrin to which it is bound. This has not been considered previously in analysis of thrombolysis. We found that tPA is forced to unbind about half the time, depending on the conditions. Since the ease with which tPA moves through a clot is an important determinant of lysis speed, tPA’s forced unbinding by plasmin could serve to increase clot lysis rates. On the other hand, such-released tPA might travel elsewhere in the vasculature and contribute to bleeding. Currently, the macroscale model does not distinguish between forced-unbound or kinetically-unbound tPA when allowing tPA to rebind to the clot. In the future, we may allow forced-unbound tPA to diffuse (as part of the fibrin degradation product to which it is bound), but not bind to a new fiber until it kinetically unbinds from the fibrin fragment.

The coordinated modeling and experimental studies reveal the number of tPA molecules per volume in thrombolytic therapy and provide further rationalization for the transverse cutting of fibrin fibers by plasmin. In general, only total amounts and molar concentrations of tPA have been considered previously in planning thrombolysis. Dosing regimens vary but often involve 100 mg over 2 hours. Concentrations in the plasma have been measured at about 2 *μ*g/ml, which is about 25 nM^35^ and corresponds to only 15 tPA molecules/*μ*m^3^. The 70 pM physiological tPA concentration equals only 0.04 tPA molecules/*μ*m^3^. Assuming a pore size between fibers of 1 *μ*m, the volume enclosed by nearest-neighbor fibers is on the order of 1 *μ*m^3^. This observation could partially explain why fibers are cut laterally, rather than uniformly degraded^3, 4^. Plasmin creation and activity occurs on a narrow segment of a fiber, nearby to where the tPA molecule binds; the tPA molecule facilitates local creation of plasmin, but does not contribute to plasmin production along the fiber length. Experiments showing “clusters” of plasminogen at the interface of a digesting clot^7, 36^, further support the idea that plasmin activity occurs in discrete locations. Due to tPA’s strong binding to fibrin, the local tPA concentration on fibrin fibers at the clot front might exceed 25 nM, but because there is a discrete number of tPA molecules binding to a given fiber, plasmin is generated only in local regions on the fiber, and the fiber is still likely to be cut laterally.

Fibers in clots are only about 20% protein^17, 18^, suggesting that there are large spaces between the protofibrils. However, we have shown that, even with large amounts of solvent in the fibers, protofibrils on average are only about 5 nm apart. This means that most macromolecules, for example plasminogen, which is approximately 9-11 nm in diameter^25, 26^, cannot diffuse through a single fiber since the space between protofibrils is too small. Even if protofibril spacing is wide enough for a plasminogen molecule to enter in one region of the fiber, protofibrils must be packed more closely together elsewhere, effectively trapping the plasminogen molecule in the fiber. In either situation, plasminogen is unable to freely diffuse through a single fiber. If anything, we underestimated the number of protofibrils in a fiber by overestimating the amount of fibrin protein in a single protofibril. A protofibril is likely not a solid cylinder of protein, so to maintain the measured protein percentage in the fiber, even more protofibrils would be necessary. This would further reduce the distance between protofibrils, making it even more difficult for macromolecules to diffuse through a fiber.

The possibility that plasmin can crawl across a fiber, which is consistent with the presence of multiple lysine-binding kringles, would provide a mechanism for plasmin to avoid inactivation by plasma-phase *α*2-AP. Our mathematical modeling (which makes the simplifying assumption that unbound plasmin is immediately inhibited by *α*2-AP and bound plasmin is completely protected from inhibition) defines the conditions for plasmin crawling: the rate of crawling must be much larger than the rate of unbinding from fibrin. If this is not the case, plasmin will unbind from fibrin and be inhibited by *α*2-AP before it can crawl any considerable distance. For the plasmin molecule to crawl, it would need to bind its unbound “limb” to a binding site farther along the fiber than the binding site from which it unbound. The current model counts only the number of binding events by an unbound “limb”; it does not distinguish where the “limb” binds. So, the expected number of steps before complete unbinding, ${k}_{{\rm{c}}{\rm{r}}{\rm{a}}{\rm{w}}{\rm{l}}}^{{\rm{P}}{\rm{L}}{\rm{i}}}/{k}_{{\rm{u}}{\rm{n}}{\rm{b}}{\rm{i}}{\rm{n}}{\rm{d}}}^{{\rm{P}}{\rm{L}}{\rm{i}}}$, is an overestimate. This further supports the requirement that ${k}_{{\rm{c}}{\rm{r}}{\rm{a}}{\rm{w}}{\rm{l}}}^{{\rm{P}}{\rm{L}}{\rm{i}}}$ must be much larger than ${k}_{{\rm{unbind}}}^{{\rm{PLi}}}$ if crawling is to be possible.

Crawling is merely another name for what has been called procession in the protein literature. There are several types of processive proteins. Some of them, such as molecular motors, require the energy of compounds like ATP for stepping. Others, such as polymerases, which can traverse large distances before dissociating, are processive because they largely encircle their protein or nucleic acid path. Plasmin may be processive because of multiple lysine-binding kringles, which may represent a novel mechanism to allow stepping. If indeed plasmin is processive, it may be unique in that it creates its own pathway by producing more C-terminal lysine binding sites for itself as it cleaves fibrin. Although this model suggests that crawling or procession is possible, further evidence is needed to prove that it occurs. When experimental evidence for plasmin procession is available, we can build a new model to help understand the details of crawling. The future model should include variable packing of protofibrils and spatial information about the location of plasmin binding sites and cleavage sites.

The current study is by no means exhaustive (e.g., the model does not include cells, FXIII and crosslinking, many inhibitors), but still provides valuable insight. A basic principle of mathematical modeling is to start simply and build in complexity only when necessary. A model that contains too many variables can be easily manipulated to give whatever results are desired; a simpler model is more likely to elucidate basic underlying mechanisms of the system. The model described here contains more of the known biology, structure, and biochemistry than any previous mathematical model, and is very flexible – concentrations, clot structure parameters, rate constants can be easily adjusted to study a range of values. In the future, the model can be modified to account for effects outside the scope of this paper: platelets, fibrinolytic inhibitors, FXIII activation and crosslinking, conversion from Glu- to Lys-plasminogen, tPA variants (which could suggest potential targets for new thrombolytics), etc.

While making the aforementioned modifications to the model will change the quantitative results, it is unlikely the qualitative results will be drastically different. For example, lysis front velocities will likely be slower in crosslinked clots^29, 37^, but we still expect to see coarse clots degrade faster than fine at low tPA number, and the opposite at higher tPA number; this effect is independent of the crosslinked status of the clot, and depends solely on the number of fibers at the clot front and the number of tPA molecules in the system. The number of tPA molecules per fibrin fiber at the clot front at which this switch occurs will likely be different when the model includes crosslinking (and may actually help the model better approximate the quantitative experimental values), but the qualitative effect will remain. Similarly, the conclusion that protofibril spacing in a fibrin fiber prevents the diffusion of macromolecules through a fiber should still hold under crosslinking, as crosslinking would only serve to bring the protofibrils closer together, leaving even less space for macromolecule diffusion^38^. Accounting for *α*2-AP crosslinked to the fibrin clot^37^ would likely slow down lysis and cause plasmin-mediated release of tPA to be less frequent, and would potentially impede plasmin’s ability to crawl (if *α*2-AP crosslinked to fibrin is able to inhibit bound plasmin, which is not currently known). However, all of the results presented here will still hold: low numbers of tPA molecules will result in coarse clots degrading faster than fine clots, plasmin-mediated degradation of fibrin will force bound tPA to be removed from the clot front, and for plasmin to be able to crawl, the crawling rate will need to be much larger than the unbinding rate.

Thrombolytic drugs are commonly used to treat thrombosis, including ischemic stroke. All of our new findings may have significant implications for clinical treatment of thrombosis. Understanding the effective diffusion of tPA through a thrombus, and the interplay between thrombus structure and tPA in the rate of thrombolysis, can aid in the development of more effective dosing regimens. Also, by investigating a wide range of tPA and plasminogen kinetic conditions, we can use the model to suggest promising targets for engineering novel thrombolytics. In the future, the model will be extended to include more inhibitors (plasminogen activator inhibitor-1, thrombin activatable fibrinolysis inhibitor), cellular components (platelets and red blood cells), and FXIIIa-mediated crosslinking. More direct applications to thrombolytic therapy will be possible with the extended model, but the current model still provides important insights to the underlying mechanisms of lysis.

## Electronic supplementary material

Supplementary Information
