# Supplementary material for: Molecular and Physical Mechanisms of Fibrinolysis and Thrombolysis from Mathematical Modeling and Experiments
Source: Sci Rep. 2017 Aug 7;7:6914. doi: 10.1038/s41598-017-06383-w (PMC5547096; doi:10.1038/s41598-017-06383-w)
Supplement: Supplementary file 1 — Supplementary Information [file 41598_2017_6383_MOESM1_ESM.pdf]

# Supplementary Information

## Molecular and Physical Mechanisms of Fibrinolysis and Thrombolysis from Mathematical Modeling and Experiments

Brittany E. Bannish,[1] Irina N. Chernysh,[2] James P. Keener,[3] Aaron L. Fogelson,[3] John W. Weisel[2]

[1]Department of Mathematics and Statistics, University of Central Oklahoma, Edmond, OK 73034, USA
[2]Department of Cell and Developmental Biology, University of Pennsylvania School of Medicine, Philadelphia, PA 19104, USA
[3]Departments of Mathematics and Bioengineering, University of Utah, Salt Lake City, UT 84112-0090, USA

The microscale model has two spatial dimensions and represents a single fiber cross section (Figure 1A,B), while the macroscale model is 3-dimensional and represents a full fibrin clot (Figure 1C-E). Variables included in the model are tPA, plasminogen, plasmin, and fibrin. The multiscale model tracks these variables as they change in both space and time. Below we list the major assumptions of the model, and highlight how the model differs from our previously published model[1].

1. Both the microscale and macroscale models include a plasma phase and a fibrin phase.
2. Experimentally determined rate constants are used if available (Table 1).
3. The microscale model uses a biologically realistic fibrin concentration within a fiber (~ 800 $\mu$M) and percentage of fibrin per fiber (~ 20%). (The previous model used 2115 $\mu$M fibrin within a fiber and about 50% of the fiber was protein.)
4. The macroscale model uses a biologically realistic fibrin concentration in the clot (8.8 $\mu$M). (The previous macroscale model clot contained closer to 22 $\mu$M fibrin.)
5. The macroscale model includes physiological fiber diameters and pore sizes for clots of various structure.
6. We account for tension of the fiber by having the fiber break if 2/3 of the fibrin in the cross section has degraded.
7. The microscale model accounts for the physical size of plasminogen molecules.
8. The microscale model includes the following reactions:

   (a) tPA, plasminogen, plasmin binding to and unbinding from fibrin. The $K_d$ for plasminogen binding to fibrin is different for intact or nicked fibrin. The $K_d$ for tPA binding to fibrin is different in the presence or absence of fibrin-bound plasminogen.

   (b) activation of plasminogen to plasmin by tPA when tPA and plasminogen are co-localized on fibrin.

(c) plasmin "crawling" to adjacent binding sites. The rates of plasmin unbinding from fibrin presented in the literature range from 0.05 $s^{-1}$ to 57.6 $s^{-1}$ [2,3]. In our simulations we use $k_{\text{unbind}}^{\text{PLi}} = 0.05\ \text{s}^{-1}$ as the unbinding rate and $k_{\text{crawl}}^{\text{PLi}} = 57.6\ \text{s}^{-1}$ as the crawling rate. In order for plasmin to crawl, the rate of unbinding must be much less than the rate of crawling (see Results and Supplementary Information "plasmin crawling calculation").

(d) plasmin-mediated degradation of fibrin.

(e) exposure of new binding sites.

9. The macroscale model:

   (a) tracks tPA molecules as they diffuse through the clot, bind to fibrin and unbind from fibrin.

   (b) uses data obtained from the microscale model to determine when individual fibers degrade (tPA binding on the macroscale initiates the fibrinolytic cascade on the microscale, creating plasmin which degrades the fiber).

From the model, we know the amounts and spatial distributions of tPA, plasminogen, plasmin, and fibrin as functions of time.

## Model Modifications

**Geometry** The first major change to the model presented in Bannish, et al.[1] is an adjustment of the microscale model domain to better reflect biologically realistic fibrin volumes and concentrations within a fiber. Fibrin fibers are approximately 20% protein and 80% water and have a fibrin concentration of about 824 $\mu$M[4,5]. We use the same fibrin concentration for both thin and thick fibers. Several papers in the literature suggest this is not valid[6-8], however there is sharp disagreement on the differences in protofibril arrangement in thin and thick fibers, so we prefer to keep the model simple until there is some consensus on this point. Consider a thin fiber with diameter 97.5 nm and a thick fiber with diameter 195 nm (in Bannish et al.[1] the diameters were 100 nm and 200 nm, respectively). For the thin fiber, instead of using 15 binding locations along one row of the square cross section, we now use 9. These binding locations correspond to protofibrils. For the thick fiber we now use 18 binding locations along one row instead of 30. This results in 19.63% of the fiber being protein, and an 800.95 $\mu$M fibrin concentration per fiber:

Percent protein per fiber

$$\frac{9^2 \text{ protofibrils}}{22.5\pi(48.75)^2 \text{ nm}^3} \times \frac{22.5\pi(2.4)^2 \text{nm}^3}{1 \text{ protofibril}} = 19.63\%$$

Fibrin concentration per fiber

$$\frac{9^2 \text{ monomers}}{22.5\pi(48.75)^2 \text{ nm}^3} \times \frac{10^6\ \mu\text{mol}}{6.02\times10^{23} \text{ monomers}} \times \frac{10^{24} \text{nm}^3}{1 \text{ L}} = 800.95\ \mu\text{M}$$

In performing these calculations, each binding location is assumed to represent a protofibril. The calculations require volumes, so we assume that the "length" of a cross section is 22.5 nm – the distance between binding sites along a fiber. In a 22.5 nm length of protofibril there is 1 fibrin monomer, because the half-staggering arrangement of monomers results in one half of two separate fibrin monomers coming together to form the protofibril.

The new microscale domain is more accurate with respect to fibrin volume and concentration, and also with respect to protofibril spacing. When we calculated that 15 protofibrils would fit in one row of the thin fiber cross section in Bannish et al.[1], we measured from one protofibril midpoint to the neighboring protofibril midpoint, and essentially packed protofibrils very close together. The new geometry, with protofibrils spaced 4.8 nm apart, is more realistic than abutting protofibrils and results in a far more accurate protein percentage (about 20% vs. 56%).

Because the microscale model domain is different, we must also adjust the macroscale model geometry. We take the fibrin concentration per fiber to be 800.95 $\mu$M and the fibrin concentration averaged over the clot to be 8.8 $\mu$M[9]. Given a fixed fiber diameter and assuming the clot is formed in a small chamber with dimensions $100\,\mu\text{m} \times 100\,\mu\text{m} \times \text{depth}\,\mu\text{m}$, we find the number of lattice nodes and pore size necessary to keep these concentrations fixed. The new number of nodes in one row of the fine (coarse) lattice is 69 (35), with pore size 1.37 $\mu$m (2.74 $\mu$m). This results in a fine (coarse) clot with 14145 (3605) fibers. To calculate the node number, $N$, notice that the number of edges in a 3-D square lattice (that is one fiber deep in one direction) is $N^2 + 2N(N-1)$ (Supplement Figure 1). To enforce concentrations, node number and pore size (p.s.) are found by solving the system of equations given below:

$$\text{Fibrin concentration in clot} = \frac{\text{volume of fibers}}{\text{volume of clot}} \times \text{fibrin concentration per fiber:}$$

$$8.8\,\mu\text{M} = \frac{[N^2 + 2N(N-1)]\pi r^2 \text{p.s.}}{100^2 \text{p.s.}} \times 800.95\,\mu\text{M}$$

$$\text{Length of lattice} = (\text{nodes in one row}) \times \text{diameter} + (\text{pores in one row}) \times \text{p.s.:}$$

$$100\,\mu\text{m} = N \times \text{diameter} + (N-1) \times \text{p.s.}$$

The final change to the macroscale model is the parameter which describes the concentration of fibrin on the fiber surface available for tPA binding, $b$. The fibrin concentration in a fiber is 800.95 $\mu$M, but not all of the fibrin protein is initially accessible for tPA binding. We define $b$ (the concentration of fibrin available for tPA binding) to be the concentration of fibrin on the surface of the fiber. The number of protofibrils on the fiber surface is the number of binding locations along the edge of the square cross section. For a thin (thick) fiber, 32 (68) of the 81 (324) protofibrils are on the surface of the fiber, or 39.51% (20.99%) of the total fibrin. This means that the fibrin concentration on the surface of a fiber available for tPA binding is $b = 316$ $\mu$M in fine clots, and $b = 168$ $\mu$M in coarse clots. As before, the time that tPA binds to a fiber in the macroscale model is calculated from an exponential distribution with rate parameter $b \cdot k^{\text{tPA}_{\text{on}}}$.

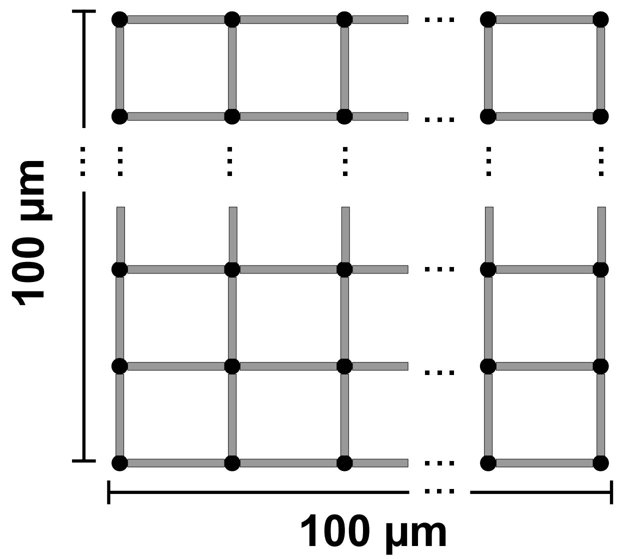


**Supplement Figure 1:** Macroscale model set up. Each black dot (or "node" of the lattice) represents a fiber coming out of the page. There are $N$ of these nodes in each row and column of the lattice, for a total of $N^2$ fibers coming out of the page. The fibers in the plane of the page are represented by the thin gray rectangles. Since there are $N$ nodes in a single row, there are $N$-1 horizontal fibers in a single row, for a total of $N(N-1)$ horizontal fibers in the clot. Similarly, there are $N(N-1)$ vertical fibers in the clot. So the total number of fibers in the clot is $N^2 + 2N(N-1)$. To calculate the value of $N$ for a given clot, we solve the equations $8.8\ \mu\mathrm{M} = \frac{[N^2+2N(N-1)]\pi r^2 \mathrm{p.s.}}{100^2 \mathrm{p.s.}} \times 800.95\ \mu\mathrm{M}$ (which says that the fibrin concentration in the clot should equal the fibrin concentration in a single fiber times the total number of fibers) and $100\ \mu\mathrm{m} = N \times \mathrm{diameter} + (N-1) \times \mathrm{p.s.}$ (which says that the length of the clot should equal the sum of the lengths of the fiber diameters and the pore sizes in one row of the lattice). In these calculations, p.s. is pore size, the distance between fibers.

**tPA activation and plasmin-mediated degradation** In the microscale model described in Bannish et al.[1], tPA could only convert plasminogen to plasmin if tPA and plasminogen were bound to the same doublet. Physiologically, however, the size of plasminogen (9-11 nm in diameter)[10] suggests that a plasminogen molecule bound anywhere in a 5-nm diameter protofibril cross section should be accessible to a tPA molecule in the same cross section. Therefore, we relax the constraint that tPA and plasminogen must share a doublet for conversion to plasmin to occur, and instead allow tPA to convert *any* plasminogen at the same binding location to plasmin. For simplicity we assume that the relative orientation of tPA and plasminogen is identical at any doublet at a given binding location, so the rate constants are the same as before, regardless of what doublet(s) the molecules inhabit. In practice, this amounts to the consideration of additional reactions in the Gillespie algorithm: now tPA can convert plasminogen on any of the 6 doublets at its location to plasmin. Similarly, we now allow plasmin to degrade any doublet at the same binding location, not just the doublet it occupies.

This implies that plasmin is large enough to reach, and degrade, any of the 6 chains of the protofibril to which it is bound. If there is more than one degradable doublet at the same binding location as plasmin, we assume the rate of degradation for each doublet is the same, so there is no bias in which doublet is chosen for degradation. Similarly, the rate of conversion of plasminogen to plasmin is the same for each plasminogen molecule at the binding location containing tPA.

**Parameter values** We adjust multiscale model parameters to reflect additional references from the literature. Table 1 contains the model parameters with the corresponding references. We have not found experimental measures of tPA and PLG binding and unbinding rates, only dissociation constants. We use binding rates from the modeling paper by Wootton, et al.[3], and choose unbinding rates to satisfy the measured dissociation constants.

**Using microscale data in the macroscale model** In Bannish, et al.[1] we described how the microscale data was incorporated in the macroscale model. Briefly, when a tPA molecule bound to a fiber in the macroscale model we generated a random number and used it with the empirical cumulative distribution function (CDF) of tPA leaving times to determine when to unbind the tPA molecule. We then found the mean number of plasmin molecules generated in the chosen tPA leaving time. Finally, we used a power function fit to a scatter plot of the lysis time vs. plasmin number data to determine the lysis time associated with the given number of plasmin molecules.

With the modifications made to the microscale model, we no longer have linear and power law relationships (between tPA leaving time and number of plasmin molecules produced, and between number of plasmin molecules produced and lysis time, respectively) to exploit in the macroscale model, so we now directly use distributions. We record the tPA leaving times and lysis times from 10,000 microscale model simulations. If lysis does not occur in a given run, a placeholder lysis time of 9000 seconds is assigned. The tPA leaving time data are sorted in ascending order, and the lysis time data are rearranged accordingly to keep the lysis times in the same position as their corresponding tPA leaving times. We represent the 10,000 tPA leaving times by 100 discrete times (corresponding to the 100 percentiles of the original 10,000 tPA leaving times) and use this data to make an empirical tPA leaving time CDF. When a tPA molecule binds to a fiber in the macroscale model, a uniformly distributed random number, $r_1$, is generated and used to interpolate a tPA leaving time from the CDF.

To determine when the fiber will degrade based on the tPA leaving time, we put the sorted 10,000 lysis times into 100 bins and select the $\lceil r_1 \times 100 - 0.5 \rceil^{\text{th}}$ bin. Sorting the entries of this bin allows us to generate an empirical lysis time CDF for the chosen tPA leaving time. We then generate a second uniformly distributed random number and use it to interpolate a lysis time from the CDF. During the interpolation, if we access a lysis time of 9000 s (the placeholder time for when lysis does not occur), then tPA does not initiate lysis. The fiber to which tPA is bound will not degrade until another tPA molecule binds and a lysis time less than 9000 seconds is chosen from the CDF.

**Calculation of the number of tPA molecules present with respect to fibrin fibers** In experiments that inject a bolus of tPA at concentration 5 nM (much higher than the plasma concentration of 70 pM), there are only 3 tPA molecules per cubic micron in the clot:

$$5\text{ nM tPA} = \frac{5\text{ nmol}}{\text{L}} \times \frac{\text{L}}{10^{15}\ \mu\text{m}^3} \times \frac{\text{mol}}{10^9\text{ nmol}} \times \frac{6.022 \times 10^{23}\text{ molecules}}{\text{mol}}$$
$$= \frac{3.011\text{ molecules}}{\mu\text{m}^3}$$

Replacing 5 nM with 0.07 nM in the above calculation shows that a physiological tPA concentration corresponds to 0.04 molecules/$\mu$m$^3$.

**Calculation of the average space between protofibrils** The following calculation can be done for any size fiber, but for concreteness, consider a 97.5-nm diameter fibrin fiber. For ease of computation, assume the fiber cross section is a square of area equal to the circular cross section (Fig. 4):

$$\text{Area} = \pi(48.75\text{ nm})^2 = 7466.19\text{ nm}^2 \approx (86.4\text{ nm}) \times (86.4\text{ nm}).$$

Since a fibrin fiber is approximately 20% protein, 20% of the cross sectional area (or 1493 nm$^2$) will be protein. The diameter of a protofibril is approximately 4.8 nm (as explained below). We assume that a protofibril is a solid cylinder of protein, so the amount of fibrin in the cross section of a single protofibril is $\pi(2.4\text{ nm})^2 = 18.09\text{ nm}^2$. This means there will be

$$\frac{1493\text{ nm}^2}{18\text{ nm}^2} \approx 82$$

protofibrils in the 97.5-nm diameter fibrin fiber. Equally spacing these protofibrils in the square cross section results in an edge-to-edge distance between protofibrils of approximately 4.8 nm (Figure 4).

The protofibril diameter is calculated from the molecular weight of fibrin (340,000 g/mole), and the fact that there is one full molecule in each 22.5 nm length of protofibril. The volume of a single protofibril can be approximated as $V$=22.5 $\pi$ r$^2$ nm$^3$/molecule = 22.5 $\pi$ r$^2 \times 10^{-21}$ cm$^3$/molecule. Also, $V = \frac{\text{MW} \times 0.73\text{ cm}^3/\text{g}}{6.02\times10^{23}}$, so setting $V = \frac{340{,}000 \times 0.73}{6.02\times10^{23}} = 22.5\,\pi\, r^2 \times 10^{-21}$ and solving for $r$ gives $r$ = 2.4 nm.

**Plasmin crawling calculation** Consider a plasmin molecule with 2 "limbs" it can use to crawl. There are 4 possible states this molecule can be in: $\hat{S}_{11}$ (both limbs are bound to fibrin), $\hat{S}_{10}$ (the first limb is bound to fibrin, the second is unbound), $\hat{S}_{01}$ (the second limb is bound to fibrin, the first is unbound), and $\hat{S}_{00}$ (both limbs are unbound) (Figure 5A). Plasmin is bound to fibrin in all states except $\hat{S}_{00}$. The molecule transitions between states according to the reaction diagram in Figure 5B, where we have grouped the $\hat{S}_{10}$ and $\hat{S}_{01}$ states into one state (called $S_{10}$), and we define $S_{00} = \hat{S}_{00}$, $S_{11} = \hat{S}_{11}$. We imagine that the plasmin molecule crawls by unbinding one limb and rebinding it elsewhere, while keeping the other limb bound, so $S_{10}$ represents the states from which plasmin will either step (bind the unbound limb) or unbind completely (unbind the bound limb). We are interested in the mean exit time (i.e., the time before plasmin totally unbinds and enters the $S_{00}$ state), and the mean number of transitions that can be completed in that time. We leave out the possibility of $S_{00}$ to $S_{10}$ transitions since we want to know the number of transitions that occur before the plasmin molecule reaches the $S_{00}$ state. The rate of transitioning from $S_{11}$ to $S_{10}$ is $k_-$, the rate of transitioning from $S_{10}$ to $S_{11}$ is $k_+$, and the rate of transitioning from $S_{10}$ to $S_{00}$ is $l_-$.

In the model depicted in Figure 5B, we assume that transition from $S_{10}$ to $S_{11}$ is an

attempted crawling step. We say "attempted" because this model cannot tell us *where* the limb bound, only that it did bind. A true step would be one in which the limb bound to a different site than that from which it unbound. So the mean number of transitions (i.e., steps) between states $S_{10}$ and $S_{11}$ obtained with this model will be an overestimate of the number of steps, since not all these transitions will result in a true crawling step. There must be a large number of transitions before total unbinding (i.e., before reaching state $S_{00}$) in order for a plasmin molecule to crawl. We calculate the mean number of transitions as follows:

Assume the plasmin molecule is in state $S_{10}$, since this is the most qualitatively interesting state (it represents the state from which a bound plasmin molecule can transition to an unbound molecule). We are interested in the mean number of transitions between states $S_{10}$ and $S_{11}$ before complete unbinding. A molecule in state $S_{11}$ can only transition to $S_{10}$, so assuming the molecule starts in state $S_{10}$ could underestimate the number of transitions by 1 (by not considering the first transition from $S_{11}$ to $S_{10}$) if the molecule actually started in state $S_{11}$. The probability the first transition from $S_{10}$ is to $S_{11}$ is $\frac{k_+}{k_+ + l_-}$, and the probability the first transition is to $S_{00}$ is $1 - \frac{k_+}{k_+ + l_-} = \frac{l_-}{k_+ + l_-}$. However, several transitions between states $S_{10}$ and $S_{11}$ may occur before plasmin unbinds (reaches state $S_{00}$). Let $N$ be the number of times plasmin transitions to $S_{11}$ before unbinding and calculate the probability distribution for $N$,

$$P[N = n] = \underbrace{\left(\frac{k_+}{k_+ + l_-}\right)^n}_{\substack{\text{prob. there} \\ \text{are } n\ S_{10} \to S_{11} \\ \text{transitions}}} \underbrace{\left(1 - \frac{k_+}{k_+ + l_-}\right)}_{\substack{\text{prob. of } S_{10} \to S_{00} \\ \text{transition}}}.$$

Let $A = \frac{k_+}{k_+ + l_-}$, so $P[N = n] = A^n(1 - A)$ is the probability of n transitions before unbinding. Then the expected number of transitions before unbinding is

$$E = \sum_{n=1}^{\infty} nA^n(1 - A) = A(1 - A)\sum_{n=1}^{\infty} nA^{n-1} = A(1 - A)\frac{d}{dA}\left(\frac{1}{1 - A}\right) = \frac{A}{1 - A} = \frac{k_+}{l_-},$$

where we have used the definition of the Maclaurin series of 1/(1-A) and the fact that power series with a nonzero radius of convergence can be differentiated term by term.

The intrinsic plasmin unbinding rate from fibrin is $l_-$. We see that this intrinsic unbinding rate (or the rate of one limb unbinding when one is already unbound) must be small in comparison to $k_+$ (the rate of one limb binding when the other is bound) in order for plasmin to make many crawling steps. If the rates are similar, then $k_+ \approx l_-$ and the molecule only takes 1 step, on average. The rate $l_-$ is comparable to the plasmin unbinding rate that we use in the microscale model ($k_{\text{unbind}}^{\text{PLi}}$), and $k_+$ can be thought of as a crawling rate ($k_{\text{crawl}}^{\text{PLi}}$). Therefore, if we take $k_{\text{unbind}}^{\text{PLi}} = 0.05\ \text{s}^{-1}$ and $k_{\text{crawl}}^{\text{PLi}} = 57.6\ \text{s}^{-1}$, we satisfy the condition $l_- \ll k_+$ and are guaranteed that plasmin will actually crawl. A final note: We see that $k_-$ (the rate of one limb unbinding when both are bound) does not affect how many steps occur, but will affect how long it takes a step to occur. That is, $k_-$ affects travel time, not distance traveled.

## Supplementary Information References


[1] Bannish BE, Keener JP, Fogelson AL. Modelling fibrinolysis: a 3D stochastic multiscale model. *Math Med Biol* 2014; **31**(1):17-44.

[2] Kolev K, Tenekedjiev K, Komorowicz E, Machovich R. Functional evaluation of the structural features of proteases and their substrate in fibrin surface degradation. *J Biol Chem* 1997; **272**(21):13666-13675.

[3] Wootton DM, Popel AS, Alevriadou BR. An experimental and theoretical study on the dissolution of mural fibrin clots by tissue-type plasminogen activator. *Biotechnol Bioeng* 2002; **77**(4):405-419.

[4] Carr ME, Hermans J. Size and density of fibrin fibers from turbidity. *Macromolecules* 1978; **11**(1):46-50.

[5] Voter WA, Lucaveche C, Erickson HP. Concentration of protein in fibrin fibers and fibrinogen polymers determined by refractive index matching. *Biopolymers* 1986; **25**:2375-2384.

[6] Li W, Sigley J, Pieters M, Carlisle Helms C, Nagaswami C, Weisel JW, Guthold M. Fibrin fiber stiffness is strongly affected by fiber diameter, but not by fibrinogen glycation. *Biophys J* 2016; **110**(6):1400-1410.

[7] Domingues MM, Macrae FL, Duval C, McPherson HR, Bridge KI, Ajjan RA, Ridger VC, Connell SD, Philippou H, Ariëns RAS. Thrombin and fibrinogen γ' impact clot structure by marked effects on intrafibrillar structure and protofibril packing. *Blood* 2016; **127**(4):487-495.

[8] Yeromonahos C, Polack B, Caton F. Nanostructure of the fibrin clot. *Biophys J* 2010; **99**(7):2018-2027.

[9] Diamond SL, Anand S. Inner clot diffusion and permeation during fibrinolysis. *Biophys J* 1993; **65**:2622-2643.

[10] Xue Y, Bodin C, Olsson K. Crystal structure of the native plasminogen reveals an activation-resistant compact conformation. *J Thromb Haemost* 2012; **10**:1385-1396.
